# The Effects of *Prunus spinosa* L. Flower Extracts, Model Polyphenols and Phenolic Metabolites on Oxidative/Nitrative Modifications of Human Plasma Components with Particular Emphasis on Fibrinogen In Vitro

**DOI:** 10.3390/antiox10040581

**Published:** 2021-04-09

**Authors:** Anna Marchelak, Joanna Kolodziejczyk-Czepas, Paulina Wasielewska, Pawel Nowak, Monika A. Olszewska

**Affiliations:** 1 Department of Pharmacognosy, Faculty of Pharmacy, Medical University of Lodz, 90-151 Lodz, Poland; monika.olszewska@umed.lodz.pl; 2 Department of General Biochemistry, Faculty of Biology and Environmental Protection, University of Lodz, 90-236 Lodz, Poland; joanna.kolodziejczyk@biol.uni.lodz.pl (J.K.-C.); paulinakapusta94@wp.pl (P.W.); pawel.nowak@biol.uni.lodz.pl (P.N.)

**Keywords:** *Prunus spinosa*, human plasma, fibrinogen, hemostasis, oxidative stress, peroxynitrite, cardiovascular diseases, antioxidants, polyphenols, metabolites

## Abstract

Oxidative post-translational modifications of fibrinogen (a multifunctional blood plasma protein essential for hemostasis) are associated with the pathogenesis of cardiovascular disorders (CVDs). *Prunus spinosa* flower is a herbal medicine used in an adjuvant treatment of CVDs and rich in polyphenolic antioxidants. In the present study, phytochemically standardized *P. spinosa* flower extracts, their primary native polyphenols and potential phenolic metabolites were evaluated in vitro for their protective effects on fibrinogen (isolated and in the human plasma matrix) using a panel of complementary methods (SDS-PAGE, western blot, C-ELISA, fluorometry, FRAP, TBARS). The results revealed that the tested analytes at in vivo relevant levels (1–5 µg/mL) considerably reduced the structural changes in the fibrinogen molecule under the oxidative stress conditions induced by peroxynitrite. In particular, they diminished the oxidation and/or nitration of amino acid residues, including tyrosine and tryptophan, as well as the formation of high molecular weight aggregates. The decrease in the levels of 3-nitrotyrosine was about 13.5–33.0% and 58.3–97.1% at 1 µg/mL and 50 µg/mL, respectively. The study indicated that low molecular weight polyphenols were crucial for the protective activity of the extracts toward fibrinogen and other human plasma components. The investigated model compounds effectively protected total plasma proteins and lipids against oxidative damage (by reducing the levels of 3-nitrotyrosine and thiobarbituric acid-reactive substances and normalizing/enhancing the non-enzymatic antioxidant capacity of plasma). The work provides insight into the role of native and metabolized polyphenols as contributory factors to the systemic activity of blackthorn flower extracts within the circulatory system.

## 1. Introduction

The initiation and progression of cardiovascular diseases (CVDs) is closely interconnected with the process of oxidative stress, occurring when the production of reactive oxygen species (ROS) overwhelms the antioxidant defense system. Despite ROS being generated as an unavoidable consequence of oxygen metabolism and playing a pivotal role in cellular signaling, the prolonged exposure to excess amounts of ROS might prompt severe damage to all cellular biomolecules, which results in their dysfunction and interferes with the signaling pathways [1,2,3]. Proteins are a major target for oxidative modification due to their abundance in biological systems and high rate constants for reactions with many ROS [4]. Among blood plasma proteins, fibrinogen appears to be one of the most susceptible to oxidative modifications. This multifunctional molecule plays an essential role in hemostasis and other intra- and extravascular processes. Oxidative stress might result in different structural changes of the fibrinogen molecule (nitration and oxidation of tyrosine residues, formation of carbonyl groups and tryptophan residues modification), as well as distinct functional consequences (increased initial velocity of fibrin clot formation, modified fibrin clot architecture, elevated fibrin clot stiffness and decreased rate of clot lysis) [5,6,7]. Clinical studies have been consistently showing a link between fibrinogen damage, an abnormal fibrin clot structure and CVDs, including coronary artery disease, venous thromboembolism, stroke and peripheral arterial disease [8,9].

In recent years, polyphenols and polyphenol-rich plant extracts have attracted increased attention as antioxidant health-promoting agents useful in the prophylaxis and adjuvant therapy of CVDs. Numerous mechanisms have been proposed by which polyphenols might regulate redox homeostasis in living cells, protect cell constituents against oxidative damage and limit the risk of oxidative stress-related diseases including CVDs—they range from direct ROS scavenging to the role of polyphenols as signaling molecules able to stimulate the endogenic antioxidant system [10,11]. Among these mechanisms, the protective effects on fibrinogen have rarely been investigated to date [12,13], and a search for new polyphenols and plant extracts with the relevant activity is required.

*Prunus spinosa* L. (blackthorn or sloe) flower is a traditional herbal medicine valued in Central and Eastern Europe for its vasoprotective, anti-inflammatory, diuretic, detoxifying (blood purifying) and spasmolytic properties, and is recommended, among others, in the treatment of intestinal and respiratory tract disorders, as well as various CVDs, such as atherosclerosis, myocarditis and cardiac neurosis [14,15,16,17]. The results of our previous phytochemical and in vitro studies demonstrated that *P. spinosa* flowers are a rich source of natural polyphenols with strong antioxidant and anti-inflammatory activity [18,19,20,21]. In particular, it was revealed that the blackthorn extracts are able to protect human plasma components (both proteins and lipids) against modifications induced by peroxynitrite (ONOO^−^), one of the most powerful oxidative/nitrative species in vivo [18]. As the strong effects were revealed toward 3-nitrotyrosine (3-NT), a biomarker of protein damage [18], the blackthorn extracts are good candidates for testing the fibrinogen protective ability.

Plant extracts are multicomponent, and their rational use in phytotherapy requires a reliable identification of the individual constituents primarily responsible for their biological activity. Recently, more than 50 compounds representing different polyphenolic classes, including flavonols (kaempferol and quercetin glycosides), A-type proanthocyanidins and phenolic acids, have been found in blackthorn flowers [18], but there is still missing information regarding the contribution of individual phenolic compounds to the antioxidant effects of the flower extracts in plasma, including their impact on fibrinogen.

After oral administration, plant polyphenols may undergo intestinal transformations by enterocyte enzymes and microbiota and enter the bloodstream in a modified form. Consequently, the total biological effects of polyphenols in vivo can be both due to the native compounds (if absorbed unmodified) and to their metabolites formed in the intestine [22,23,24,25]. Our previous in vitro study on scavenging the most common in vivo relevant oxidants, including ONOO^−^, revealed that the phenolic metabolites, which might be produced in humans after the ingestion of blackthorn flowers, showed a significant and often stronger antioxidant capacity than their parent compounds and should not be omitted during the bioactivity assessment of the title plant [19].

Therefore, the objective of the present in vitro study was (i) to evaluate the potential protective effects of the blackthorn flower extracts on fibrinogen under oxidative stress conditions induced by ONOO^−^, (ii) to assess the contribution of individual model native polyphenols to the extracts’ antioxidant activity in plasma, including their effects on fibrinogen, and (iii) to compare the activity of native compounds with that of their potential metabolites. The changes in the fibrinogen structure were studied using complementary qualitative and quantitative methods (SDS-PAGE electrophoresis, western blot, the competitive ELISA and fluorometric assays). The antioxidant effects in plasma were tested by the measurements of the non-enzymatic antioxidant capacity of plasma (NEAC) and the levels of 3-NT and thiobarbituric acid-reactive substances (TBARS). The plant extracts for the tests were obtained during our previous study [18] and characterized in detail using a panel of phytochemical profiling methods including LC-MS/MS and LC-PDA assays, fully validated for the quantitative purposes [18,20,21].

## 2. Materials and Methods

### 2.1. Plant Material and Extracts Preparation

The analyses were performed using the source hydroalcoholic (methanol-water, 7:3, *v*/*v*) extract (MED) and its diethyl ether (DEF) and ethyl acetate (EAF) fractions obtained previously by fractionated extraction from the flowers of *P. spinosa* L. [18]. The commercially available sample of the plant material was purchased in 2015 (harvest in April 2015) from Dary Natury (Koryciny, Poland). The preparation of the extracts and their phytochemical standardization using comprehensive UHPLC-MS/MS, HPLC-PDA and spectrophotometric profiling were described previously [18,20,21]. The quantitative profile of the extracts is presented in Table 1 and Table S1.

### 2.2. Reference Standards of Model Polyphenols and Phenolic Metabolites

**Figure 1:**
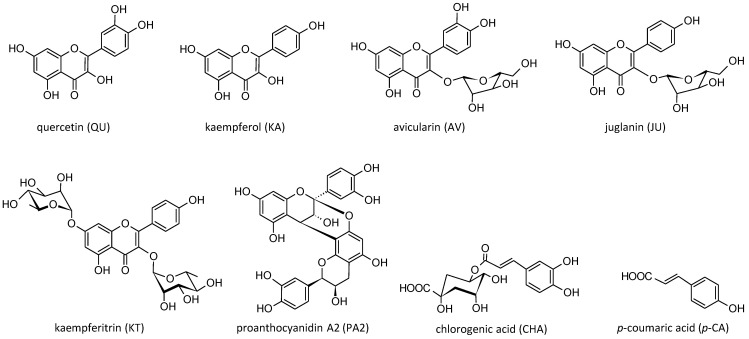
Structures of the investigated model native polyphenols of *P. spinosa* flower extracts.

**Figure 2:**
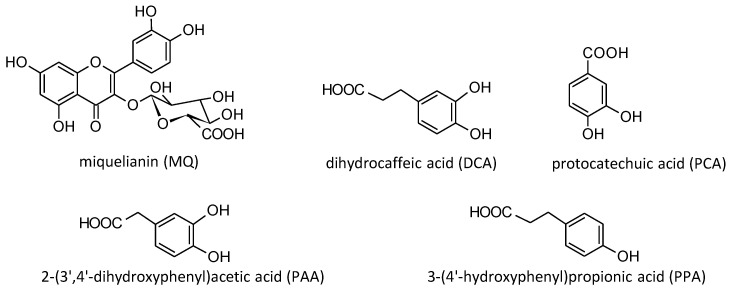
Structures of the investigated phenolic metabolites.

High-purity standards, such as chlorogenic acid (5-*O*-caffeoylquinic acid, CHA); quercetin (QU); kaempferol (KA); *p*-coumaric acid (*p*-CA); miquelianin (quercetin 3-*O*-β-D-glucuronopyranoside, MQ); 2-(3′,4′-dihydroxyphenyl)acetic acid (PAA); protocatechuic acid (PCA); 3-(4′-hydroxyphenyl)propionic acid (PPA); 3-(3′,4′-dihydroxyphenyl)propionic acid (dihydrocaffeic acid, DCA); and ascorbic acid (AA) were purchased from Sigma-Aldrich (St. Louis, MO, USA), while proanthocyanidin A2 (PA2) was obtained from Phytolab (Vestenbergsgreuth, Germany). The standards of juglanin (kaempferol 3-*O*-*α*-L-arabinofuranoside, JU), avicularin (quercetin 3-*O*-*α*-L-arabinofuranoside, AV) and kaempferitrin (kaempferol 3,7-di-*O*-*α*-L -rhamnopyranoside, KT) were isolated previously in the Department of Pharmacognosy, Medical University of Lodz, Lodz, Poland, from the flowers and leaves of *P. spinosa*, with HPLC and NMR purity > 98% [26,27]. The structures of the model native polyphenols and phenolic metabolites investigated in the study are presented in Figure 1 and Figure 2, respectively.

### 2.3. Synthesis of ONOO^−^

ONOO^−^ was synthesized according to a method described by Pryor et al. [28]. The synthesis was based on the reaction of ozone with azide ions under alkaline conditions (pH 12). It yields the peroxynitrite concentration up to 70–80 mM with low ionic strength and eliminates the risk of contamination by hydrogen peroxide. The product concentration was estimated spectrophotometrically at 302 nm, using the molar absorption coefficient of 1670 M^−1^ cm ^−1^.

### 2.4. Antioxidant Activity in Human Plasma Model

#### 2.4.1. Isolation of Blood Plasma and Preparation of Samples

The human blood plasma used for the experiments was obtained from commercially available fresh buffy coat units that were purchased from the Regional Centre of Blood Donation and Blood Treatment in Lodz (Poland). Blood was derived from healthy, non-smoking volunteers declaring a balanced diet free of antioxidant supplements. It was collected on a citrate/phosphate/dextrose (CPD) solution using the Fresenius–Kabi Compoflex system.

Plasma was obtained by the differential centrifugation of the buffy coats according to previously described procedures [29]. The study complies with the requirements of the Declaration of Helsinki and was approved by the Committee on the Ethics of Research at the Medical University of Lodz (RNN/213/18/KE) and University of Lodz (8/KBBN-UŁ/II/2015). Plasma samples were diluted with a (Ca^2+^)-free phosphate-buffered saline (PBS) purchased from Biomed (Lublin, Poland), pre-incubated for 5 min at 37 °C with the tested analytes at the final concentration of 1, 5 and 50 μg/mL, and then exposed to ONOO^−^ at the concentration of 150 μM (the FRAP assay) or 100 µM (all other experiments on plasma and isolated fibrinogen) for 5 min. Plasma samples prepared analogously but not treated with the analytes and/or ONOO^−^ constituted the control. In the case of the samples not treated with ONOO^−^ (plasma and the analytes only), there were no significant differences (*p* > 0.05) between the levels of the investigated oxidative stress markers in the plasma samples incubated with the analytes and the control (untreated) plasma. The bicinchoninic acid (BCA) assay, with the use of Pierce BCA Protein Assay Kit (Thermo Scientific, Waltham, MA, USA), was carried out to evaluate the levels of plasma proteins. In the experiments, 96-well plates and a microplate spectrophotometer, SPECTROstar Nano (BMG Labtech, Ortenberg, Germany), were used.

#### 2.4.2. Determination of 3-NT in Plasma Proteins

The 3-NT-containing proteins in blood plasma (control plasma or plasma with the analytes and exposed to ONOO^−^) were detected using the competitive enzyme-linked immunosorbent assay (C-ELISA) carried out analogously to the previous procedure [29], using the Abcam (Cambridge, UK) immunoreagents. The levels of 3-nitrotyrosine in plasma proteins were quantified based on the standard curve, prepared from nitrated standard (i.e., fibrinogen) solutions. The data were presented as the 3NT-Fg equivalents (in nanomoles per mg of plasma protein).

#### 2.4.3. Determination of TBARS in Plasma

The evaluation of the TBARS generation in the examined samples (control plasma or plasma with the analytes and exposed to ONOO^−^) was performed analogously to procedures described in our previous work [29]. The reagents required for this assay, including 2-thiobarbituric acid, were obtained from Sigma-Aldrich (St. Louis, MO, USA).

#### 2.4.4. Determination of the NEAC of Plasma

The effects of the examined analytes on the NEAC of blood plasma were evaluated in the FRAP assay by measuring the ability of the tested samples to reduce ferric to ferrous ions, according to the previously described procedure [18]. The NEAC values correspond to the non-enzymatic antioxidant status of blood plasma, which depends on low molecular antioxidants present in this biological fluid, including both endogenous and diet-derived antioxidants such as polyphenols and their metabolites [30]. The FRAP of the assayed samples (control plasma and plasma with the analytes and exposed to ONOO^−^) was quantitatively expressed as millimolar equivalents of ferrous (Fe^2+^) ions. The calibration curve was prepared using different concentrations of ferrous sulphate. All reagents were purchased from Sigma-Aldrich (St. Louis, MO, USA).

### 2.5. Activity against Oxidative/Nitrative Modifications of Human Fibrinogen

#### 2.5.1. Isolation of Fibrinogen from Blood Plasma

Fibrinogen was isolated from human plasma (obtained as described in Section 2.4.1) by the cold ethanol precipitation technique according to Doolittle, as described previously [6]. The fibrinogen concentration was established spectrophotometrically at 280 nm using an extinction coefficient 1.55 for 1 mg/mL solution.

#### 2.5.2. SDS PAGE Analysis

Fibrinogen was pre-incubated for 20 min at room temperature with the examined analytes, added to the final concentration range of 1–50 μg/mL and then exposed to 100 μM of ONOO^−^ for 5 min. The samples were subsequently diluted in a ratio of 1:1 with the reducing agent containing 950 µL 2x Laemmli sample buffer (Bio-Rad, Hercules, CA, USA) and 50 µL 2-mercaptoethanol (Sigma-Aldrich, St. Louis, MO, USA), and heated at 95 °C for 5 min. The final concentration of fibrinogen was 1 µg/mL. Control samples were prepared analogously, using fibrinogen not treated with the analytes and/or ONOO^−^.

Samples were separated on SDS-PAGE using a Mini-Protean Electrophoresis Cell (Bio-Rad, Hercules, CA, USA) and 4–20% Mini-PROTEAN TGX Precast Gels (Bio-Rad, Hercules, CA, USA). A running buffer was prepared by adding 100 mL 10x Tris/Glycine/SDS running buffer (Bio-Rad, Hercules, CA, USA) to 900 mL deionized water. The protein was stained with the mixture of 0.125% (*m*/*v*) Coomassie Brilliant Blue R250 (Thermo Scientific™, Waltham, MA USA), 50% (*v*/*v*) methanol, 10% (*v*/*v*) acetic acid and deionized water. After staining, the Coomassie dye was removed by aspiration, and the gels were covered with the destaining solution, containing 25% (*v*/*v*) methanol, 10% (*v*/*v*) acetic acid and deionized water. The destaining solution was changed several times, and the destaining was continued until the protein bands were seen without background staining of the gel. A densitometric analysis of the obtained gels was performed with CLIQS Gel Image Analysis Software (TotalLab, Newcastle-Upon-Tyne, UK).

#### 2.5.3. Western Blot Analysis

Western blot analysis was conducted in the isolated fibrinogen and plasma samples. For western blot, SDS-PAGE was performed using 4–15% Mini-PROTEAN TGX Precast Gels (Bio-Rad, Hercules, CA, USA), as described in Section 2.5.2, skipping the staining and destaining steps. The electrophoretic transfer onto the Immobilon^®^-P polyvinylidene fluoride (PVDF) membrane (Sigma-Aldrich, St. Louis, MO, USA) was performed using the Mini Trans-Blot Cell (Bio-Rad, Hercules, CA, USA). The membrane was activated by wetting in 100% (*v*/*v*) methanol for 5 s, and then rinsed with deionized water. The transfer buffer (pH 8.3) was prepared by mixing 25 mM Tris, 192 mM glycine, 20% (*v*/*v*) methanol, 0.05% (*m*/*v*) SDS and deionized water. The running conditions were as follows: 4 °C, 30 V, constant 90 mA and overnight transfer in a Mini Trans-Blot Module (Bio-Rad, Hercules, CA, USA).

In the case of the fibrinogen samples, the membrane was blocked for 1.5 h with a 5% (*m*/*v*) defatted dry milk solution in a mixture of 10 mM Tris/HCl, 150 mM NaCl, 0.05% (*v*/*v*), Tween and deionized water (TBS-T) (pH 7.4), and then incubated for 1 h with an anti-3-NT goat polyclonal antibody (Abcam, Cambridge, UK) diluted 1:20,000 in a 5% (*m*/*v*) non-fat dry milk solution in TBS-T. Each membrane was washed six times for 5 min with TBS-T, and next incubated for 1 h with the monoclonal anti-goat/sheep IgG-peroxidase antibody (Sigma-Aldrich, St. Louis, MO, USA) diluted 1:500 in TBS-T with a 5% (*m*/*v*) solution of defatted milk. Each blot was washed six times for 5 min with TBS-T. The bands were visualized by a luminol-enhanced chemiluminescence (ECL) system and exposed to X-ray film.

In the case of plasma samples, the membrane was blocked 1.5 h with 5% (*m*/*v*) defatted dry milk solutions in TBS-T and incubated for 1 h with an anti-fibrinogen antibody produced in goat (Sigma-Aldrich, St. Louis, MO, USA) diluted 1:6000 in 5% (*m*/*v*) defatted milk solutions in TBS-T. Each membrane was washed six times for 5 min with TBS-T, and then incubated for 1 h with the rabbit anti-goat IgG (H+L) secondary antibody (Thermo Scientific™, Waltham, MA USA), diluted 1:5000 in TBS-T with a 5% (*m*/*v*) solution of defatted milk. Each blot was washed six times for 5 min with TBS-T. The bands were visualized by a luminol-enhanced chemiluminescence (ECL) system and exposed to X-ray film.

The blot images were analyzed using CLIQS Gel Image Analysis Software (TotalLab, Newcastle-Upon-Tyne, UK). For molecular weight (MW) estimation on SDS-PAGE and the western blot analyses, the Precision Protein Plus Dual Color standards (Bio-Rad, Hercules, CA, USA) were employed. The MW of an unknown band was determined in the following way: a standard curve of the log (MW) versus Rf (the ratio of the migration distance of the protein to the migration distance of the dye front) was generated using the standards. A strong linear relationship (*r* > 0.99) between the MWs of the proteins and migration distances was observed, which confirmed the reliability in predicting MW.

#### 2.5.4. C-ELISA of the ONOO^−^-Induced 3-NT Formation

The immunodetection of the 3-NT-containing protein in the samples was carried out using the C-ELISA. The assayed samples included the control fibrinogen and the fibrinogen treated with the analytes and ONOO^−^ (as described in Section 2.4.2). The nitrated proteins levels were quantified based on a standard curve, prepared with the use of different concentrations of a nitrated fibrinogen. The obtained results are presented in nanomoles of 3NT-Fg equivalents per mg of plasma protein.

#### 2.5.5. Fluorometric Analysis of the ONOO^−^-Induced Tryptophan Residue Modifications

The assay was based on measurements of the fluorescence of tryptophan residues. The indole group of this amino acid is responsible for UV-induced excitation and a source of emission [31]. The exposure of the native protein (e.g., fibrinogen) to ONOO^−^-induced oxidative stress results in the oxidation and/or nitration of the tryptophan molecule, leading to a decline of its fluorescence. The examined protein samples (2 mg/mL, in 0.1 M Tris/HCl, pH 7.4; control fibrinogen and the ONOO^−^-treated samples) were assayed using Cary Eclipse spectrofluorometer (Agilent, Santa Clara, USA) in 96-well microplates. The excitation wavelength was 280 nm, and the emission wavelength was 340 nm. The slit width was 5 nm.

### 2.6. Statistical Analysis

The results are reported as mean values ± standard error (SE) for the indicated number of experiments. The significance of the differences between means were evaluated with the use of a one-way ANOVA for repeated measurements, followed by Dunnett’s tests. The correlations were determined using an *F*-test. All calculations were performed using the Statistica 12 Pl software for Windows (StatSoft Inc., Krakow, Poland). The *p* values lower than 0.05 were considered as significant.

## 3. Results

### 3.1. Protective Effects on Human Plasma Components against the ONOO^−^-Induced Oxidative Stress

**Figure 3:**
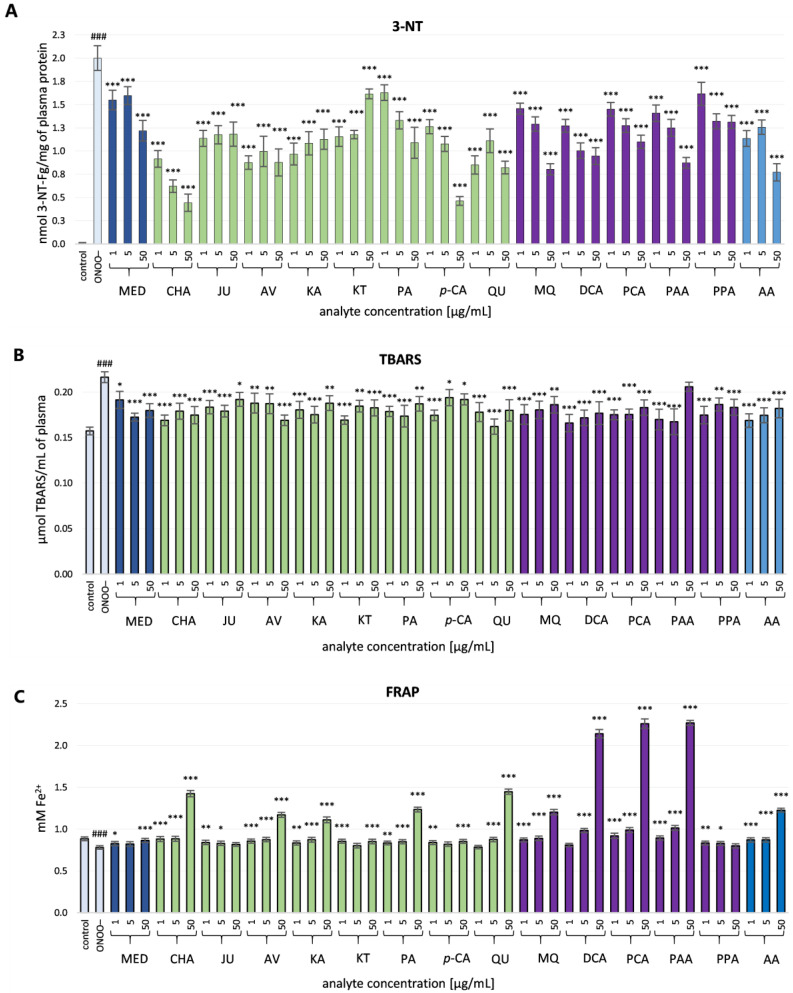
Effects of the source methanol-water extract (MED) from *P. spinosa* flower, its native model constituents and potential metabolites in vivo on human plasma exposed to oxidative stress: (**A**) effects on the nitration of tyrosine residues in plasma proteins and formation of 3-NT; (**B**) effects on the peroxidation of plasma lipids (measured as TBARS formation); (**C**) effects on NEAC (measured by FRAP). Results are presented as means ± SE (*n* = 14). Statistical differences: ### *p* < 0.001 for control plasma versus ONOO^−^-treated plasma (without the analytes); * *p* < 0.05, ** *p* < 0.01, and *** *p* < 0.001 for ONOO^−^-treated plasma in the presence of the analytes (1, 5, 50 µg/mL) versus ONOO^−^-treated plasma in the absence of the analytes. Bars colors: light blue—control and ONOO^−^-treated plasma without the analytes; navy blue—*P. spinosa* flower extracts; green—model polyphenols; violet—phenolic metabolites; blue—positive standard (AA).

The exposure of plasma samples to ONOO^−^ at the concentration of 100 or 150 μM induced substantial oxidative and nitrative damage to blood plasma components, confirmed by the measurements of well-known oxidative stress biomarkers. In comparison to the control (untreated ONOO^−^) samples, the significantly elevated levels (*p* < 0.001) of 3-NT (Figure 3A) and TBARS were observed (Figure 3B). Moreover, the ONOO^−^-treated plasma exhibited a moderate decrease (*p* < 0.001) of NEAC, as measured by the FRAP assay (Figure 3C).

In plasma samples incubated with ONOO^−^ in the presence of the investigated analytes (at 1–50 µg/mL), the rate of oxidative/nitrative modifications of the plasma components was significantly diminished (Figure 3A–C). All the tested analytes efficiently reduced the nitration of protein tyrosine residues (*p* < 0.001, Figure 3A). In the case of MED, the inhibition of 3-NT generation reached 22.5% and 39.1% at 1 µg/mL and 50 µg/mL, respectively. An Even larger decrease in the 3-NT level was found for model compounds (by about 18.6–57.5% and 19.3–77.8% at 1 µg/mL and 50 µg/mL, respectively). Native polyphenols such as CHA, AV, QU and KA inhibited the nitration of tyrosine residues (*p* < 0.001) by more than 50%, even at the concentration of 1 µg/mL. At 50 µg/mL, the highest inhibition was observed for CHA and *p*-CA (by about 77.81% and 76.86%, respectively). The investigated phenolic metabolites were also able to protect plasma proteins against 3-NT production (by about 19.2–36.4% and 34.4–59.8% at 1 µg/mL and 50 µg/mL, respectively). The activity of the positive standard AA was on a similar level, as the decrease in 3-NT was about 43.3% and 61.4% at 1 µg/mL and 50 µg/mL, respectively.

Furthermore, all of the analytes slightly lowered the ONOO^−^-induced plasma lipid peroxidation, as demonstrated by the decreased levels of TBARS by about 11.5–20.3% for MED, 11.3–21.9% for model compounds, 4.9–23.3% for metabolites and 15.9–22.1% for AA (Figure 3B). The protective effects were statistically significant (*p* < 0.05), except for PAA at 50 µg/mL; however, for most of the analytes, they were not dose-dependent, as the highest concentrations corresponded to the lowest activities.

In contrast, the ability of the analytes to normalize and/or enhance the NEAC of plasma was dose-dependent (Figure 3C). For most of the analytes, the observed effects were statistically significant (*p* < 0.05), regardless of concentration, with the exception of MED (at 5 µg/mL), JU (at 50 µg/mL), *p*CA (at 5 µg/mL), QU (at 1 µg/mL), DCA (at 1 µg/mL) and PPA (at 50 µg/mL). Among the model native compounds, CHA, AV, KA, PA2 and QU displayed the strongest activity. In the case of these constituents, the increase in NEAC amounted to about 142.2–185.3% compared to the ONOO^−^-stimulated plasma and 125.6–163.6% compared to the control (untreated) samples. Moreover, among the phenolic metabolites, the highest enhancement in NEAC was observed for PCA (at 50 µg/mL) at about 289.5% compared to the ONOO^−^-stimulated plasma and 255.7% compared to the control (untreated) samples. The improvement in NEAC was also observed for AA, and the increase at 50 µg/mL was 157.0% and 138.7% compared to the ONOO^−^-stimulated plasma and to the control (untreated) samples, respectively.

### 3.2. Protective Effects against Oxidative/Nitrative Modifications of Fibrinogen

#### 3.2.1. SDS-PAGE Analysis of the ONOO^−^-Induced Changes in the Isolated Fibrinogen

**Figure 4:**
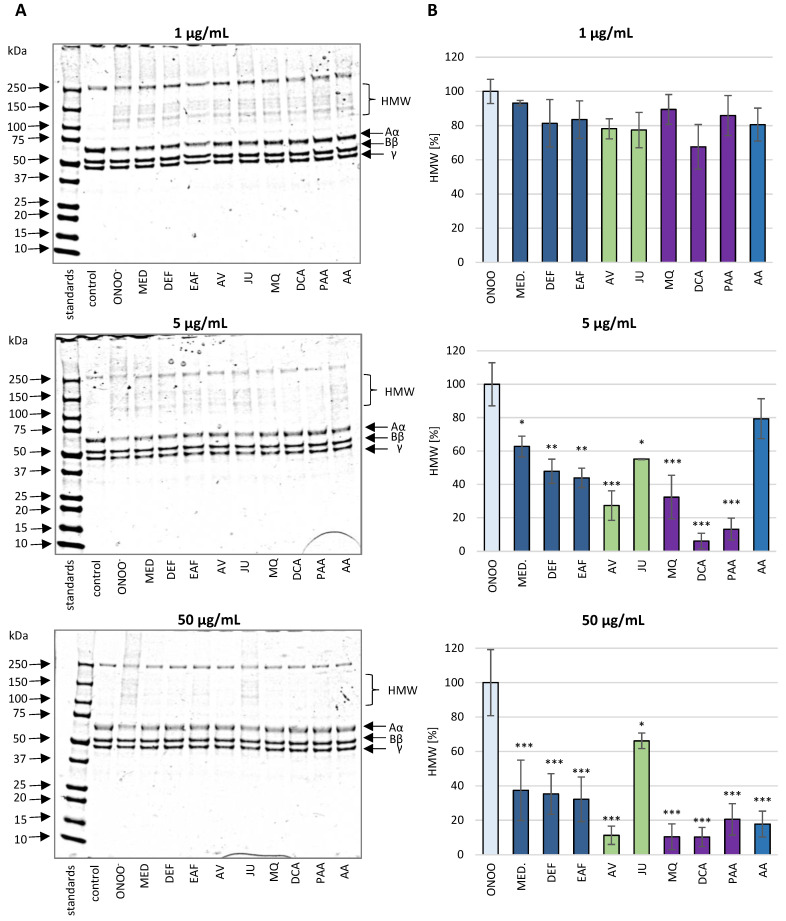
The effects of *P. spinosa* flower extracts, their native model constituents and potential in vivo metabolites on ONOO^−^-induced changes in the electrophoretic pattern of human fibrinogen. The SDS-PAGE was conducted under reducing conditions, using the 4–20% gradient gels. (**A**) The representative gels of four independent experiments; (**B**) Relative intensities of high molecular weight (HMW) aggregates’ bands detected in the fibrinogen samples. The intensities of HMW aggregates’ bands in fibrinogen treated with ONOO^−^ in the absence of the analytes was assumed as 100%. Results are presented as means ± SE (*n* = 4). Statistical differences: * *p* < 0.05, ** *p* < 0.01, and *** *p* < 0.001 for ONOO^−^-treated fibrinogen in the presence of the analytes (1, 5, 50 µg/mL) versus ONOO^−^-treated fibrinogen in the absence of the analytes. For the bars’ colors, see Figure 3.

**Figure 5:**
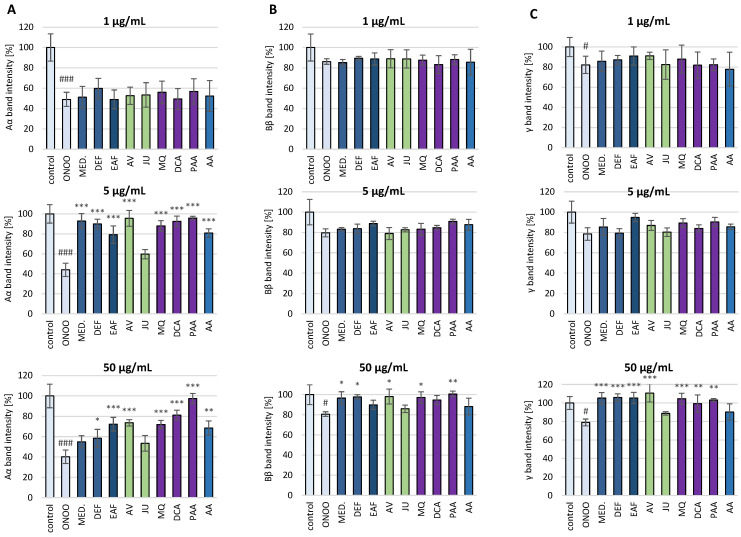
The effects of *P. spinosa* flower extracts, their native model constituents and potential in vivo metabolites on ONOO^−^-induced changes in the electrophoretic pattern of human fibrinogen: (**A**) The effects on the intensity of the band corresponding to the Aα chain; (**B**) The effects on the intensity of the band corresponding to the Bβ chain; (**C**) The effects on the intensity of the band corresponding to the γ chain. The SDS-PAGE was conducted under reducing conditions, using the 4–20% gradient gels. The Aα, Bβ and γ bands’ intensities in control fibrinogen (untreated with ONOO^−^) were assumed as 100%. Results are presented as means ± SE (*n* = 4). Statistical differences: ### *p* < 0.001 and # *p* < 0.05 for control fibrinogen versus ONOO^−^-treated fibrinogen (without the analytes); * *p* < 0.05, ** *p* < 0.01, and *** *p* < 0.001 for ONOO^−^-treated fibrinogen in the presence of the analytes (1, 5, 50 µg/mL) versus ONOO^−^-treated fibrinogen in the absence of the analytes. For the bars’ colors, see Figure 3.

SDS-PAGE analysis demonstrated that the exposure of isolated human fibrinogen to ONOO^−^ (100 µM) resulted in the oxidative stress-triggered modifications of this biomolecule (Figure 4 and Figure 5). Under the reducing conditions of SDS-PAGE, control fibrinogen samples (not treated with ONOO^−^) provided the typical electrophoretic pattern of this glycoprotein, with three bands corresponding to the Aα, Bβ and γ protein chains, with molecular weights (MWs) of 67 kDa, 56 kDa and 48 kDa, respectively. The electrophoretic pattern of fibrinogen samples treated with ONOO^−^ contained additional bands, resulting from the generation of high molecular weight (HMW) aggregates. The HMW aggregates were represented as additional bands with MW >120 kDa that were located over the typical fibrinogen chains (i.e., Aα, Bβ and γ). Furthermore, the formation of HMW aggregates was associated with lowering the intensity of the bands corresponding to the Aα, Bβ and γ chains, among which the Aα chain was the most prone to modifications (Figure 4 and Figure 5).

In the presence of the tested analytes, the oxidative modifications of the fibrinogen molecule were reduced. *P. spinosa* flower extracts, their model native constituents and polyphenolic metabolites effectively protected fibrinogen molecules against the ONOO^−^-induced modifications in a dose-dependent manner. The decrease in the intensity of the bands corresponding to the Aα chain as well as the formation of HMW aggregates was considerably reduced (Figure 4 and Figure 5).

#### 3.2.2. Western Blot Analysis of the Isolated Fibrinogen with Anti-3-NT Antibody

**Figure 6:**
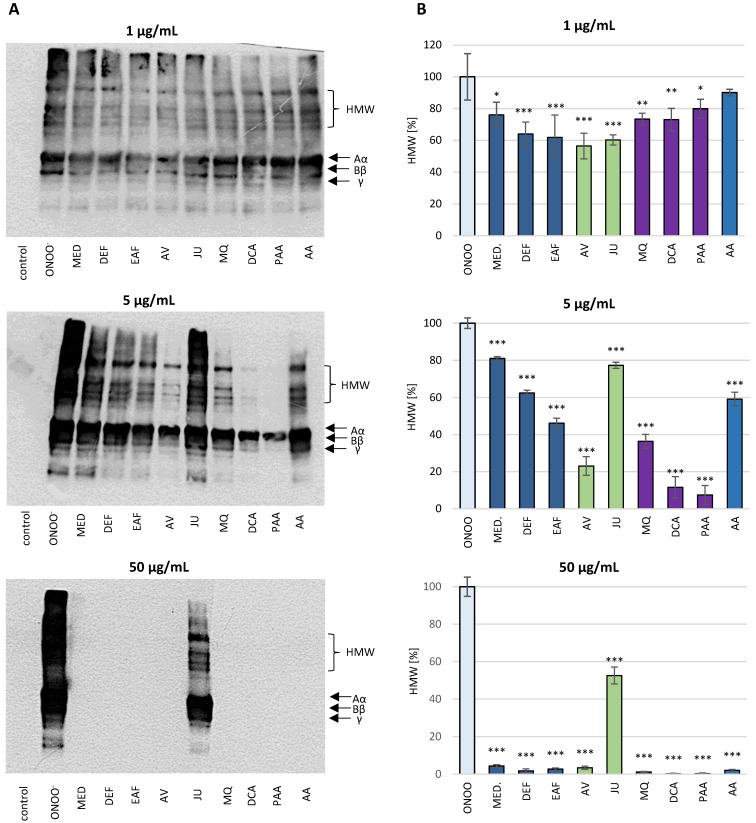
The effects of *P. spinosa* flower extracts, their native model constituents and potential in vivo metabolites on ONOO^−^-induced nitrative modifications of human fibrinogen. The western blot immunodetection of the 3-NT in the isolated fibrinogen was performed using the anti-3-NT antibody. Blots were visualized by chemiluminescence and recorded onto the X-ray films. The figure shows a typical pattern of human fibrinogen, corresponding to different isoforms of this protein, including products of the alternative splicing, post-translational modifications as well as physiological proteolysis in blood circulation (bands localized below the fibrinogen gamma chain). (**A**) The representative blots of three independent experiments. (**B**) Relative intensities of HMW aggregates’ bands detected in the fibrinogen samples. The intensities of the HMW aggregates’ bands in fibrinogen treated with ONOO^−^ in the absence of the analytes was assumed as 100%. Results are presented as means ± SE (*n* = 3). Statistical differences: * *p* < 0.05, ** *p* < 0.01, and *** *p* < 0.001 for ONOO^−^-treated fibrinogen in the presence of the analytes (1, 5, 50 µg/mL) versus ONOO^−^-treated fibrinogen in the absence of the analytes. For the bars’ colors, see Figure 3.

The nitration of tyrosine residues in the fibrinogen molecule after its exposure to ONOO^−^ was first investigated on an isolated protein by western blot analysis with the use of an anti-3-NT antibody (Figure 6). The treatment of fibrinogen with ONOO^−^ at the concentration of 100 µM induced the nitration of tyrosine residues in this protein. The presence of 3-NT in fibrinogen molecules was confirmed by the chemiluminescence of bands corresponding to the Aα, Bβ and γ chains, as well as the 3-NT-containing HMW aggregates, visible on the top of blot. In particular, the Aα chain and HMW aggregates seem to be the most intensely nitrated. Moreover, some bands localized below the γ chain could be observed, which correspond to different isoforms of fibrinogen, including products of the alternative splicing, post-translational modifications as well as physiological proteolysis in blood circulation.

The pre-incubation of fibrinogen with the investigated analytes (1–50 µg/mL) before exposure to ONOO^−^ revealed that all of them may reduce the tyrosine nitration in this molecule under the oxidative stress conditions (Figure 6). The anti-nitrative effects of the analytes were dose-dependent. At the concentration of 50 µg/mL, all of the analytes except JU almost completely inhibited 3-NT formation. The differences in the activity of individual compounds were evident at the concentration of 5 µg/mL, where the observed anti-nitrative potential of some model native compounds (AV) and metabolites (i.e., MQ, DCA and PAA) was higher than that of the positive standard AA.

#### 3.2.3. Determination of the ONOO^−^-Induced 3-NT Formation in the Isolated Fibrinogen by C-ELISA

**Figure 7:**
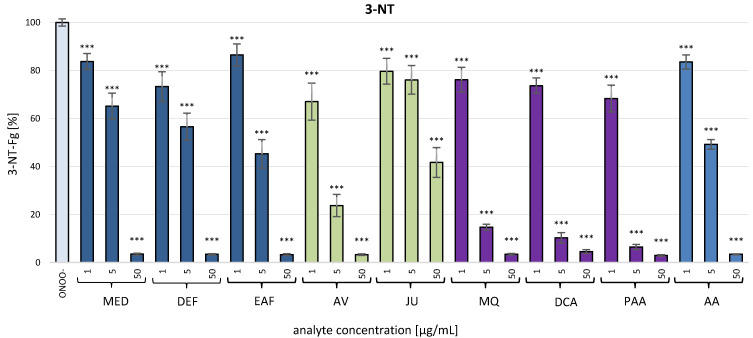
Determination of protective effects of *P. spinosa* flower extracts, their native model constituents and potential in vivo metabolites against the ONOO^−^-induced formation of 3-NT in the isolated human fibrinogen by the C-ELISA. The 3-NT level in fibrinogen treated with ONOO^−^ in the absence of the analytes was assumed as 100% of tyrosine nitration. Results are presented as means ± SE (*n* = 12). Statistical differences: *** *p* < 0.001 for ONOO^−^-treated fibrinogen in the presence of the analytes (1, 5, 50 µg/mL) versus ONOO^−^-treated fibrinogen in the absence of the analytes. For the bars’ colors, see Figure 3.

The protein exposed to ONOO^−^ in the presence of the investigated analytes (at 1–50 µg/mL) displayed a considerably reduced amount of 3-NT (*p* < 0.001; Figure 7). In the case of *P. spinosa* flower extracts, the tyrosine nitration was inhibited by about 13.5–26.7% at 1 µg/mL and over 96% at 50 µg/mL for both MED and its fractions. The model native polyphenols, AV and JU, displayed strong anti-nitrative activity with the higher inhibitory percentage found for AV (33.0% and 96.7% at 1 µg/mL and 50 µg/mL, respectively). All of the analyzed metabolites effectively protected fibrinogen against the nitration of tyrosine residues with inhibition at the level of 23.9–31.7% and 95.5–97.1% at 1 µg/mL and 50 µg/mL, respectively. Moreover, at 5 µg/mL, their inhibitory percentage was over 85%. The anti-nitrotyrosine effects of the extracts, model native compounds and phenolic metabolites were dose-dependent and comparable or stronger than that of the reference plasma antioxidant (AA), which inhibited the 3-NT formation by 16.5% and 96.6% at 1 µg/mL and 50 µg/mL, respectively.

#### 3.2.4. Determination of the ONOO^−^-Induced Modifications of Tryptophan Residues in the Isolated Fibrinogen

**Figure 8:**
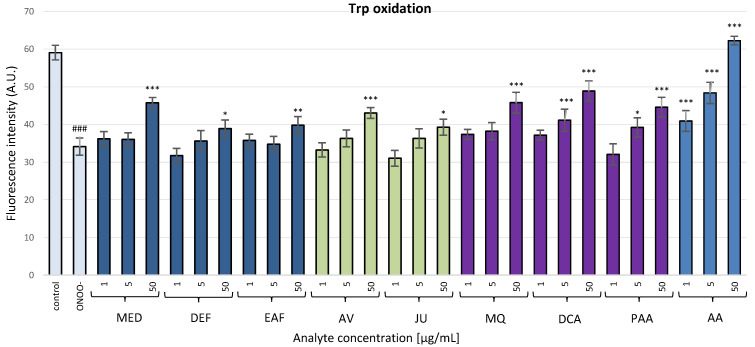
Fluorometric determination of the effects of *P. spinosa* flower extracts, their native model constituents and potential in vivo metabolites on the oxidation of tryptophan residues in human fibrinogen. The test was based on a significant decay of tryptophan fluorescence resulting from the ONOO^−^-induced oxidation of fibrinogen samples and the ability of the examined substances to prevent this effect. Results are presented as means ± SE (*n* = 8). Statistical differences: ### *p* < 0.001 for control fibrinogen versus ONOO^−^-treated fibrinogen (without the analytes); * *p* < 0.05, ** *p* < 0.01, and *** *p* < 0.001 for ONOO^−^-treated fibrinogen in the presence of the analytes (1, 5, 50 µg/mL) versus ONOO^−^-treated fibrinogen in the absence of the analytes. For the bars’ colors, see Figure 3.

In the absence of any antioxidants, ONOO^−^ induced a considerable oxidation of tryptophan residues in fibrinogen molecules (*p* < 0.001), confirmed by a 42.3% decrease in the fluorescence intensity of this amino acid. In fibrinogen samples treated with ONOO^−^ in the presence of the investigated analytes (1–50 µg/mL), some reduction of the oxidative effects of ONOO^−^ was observed (Figure 8). However, statistical significance (*p* < 0.05) was demonstrated only for the highest concentration of the extracts, model polyphenols and metabolites, except for DCA and PAA, which also considerably inhibited (*p* < 0.05) the oxidation of tryptophan residues at 5 µg/mL. The positive standard AA displayed the strongest antioxidant activity, as its effect was significant (*p* < 0.001) regardless of the concentration.

#### 3.2.5. Influence of the Analytes on the ONOO^−^-Induced Modifications of Fibrinogen in Blood Plasma Matrix—Western Blot Analysis with Anti-Fibrinogen Antibody

**Figure 9:**
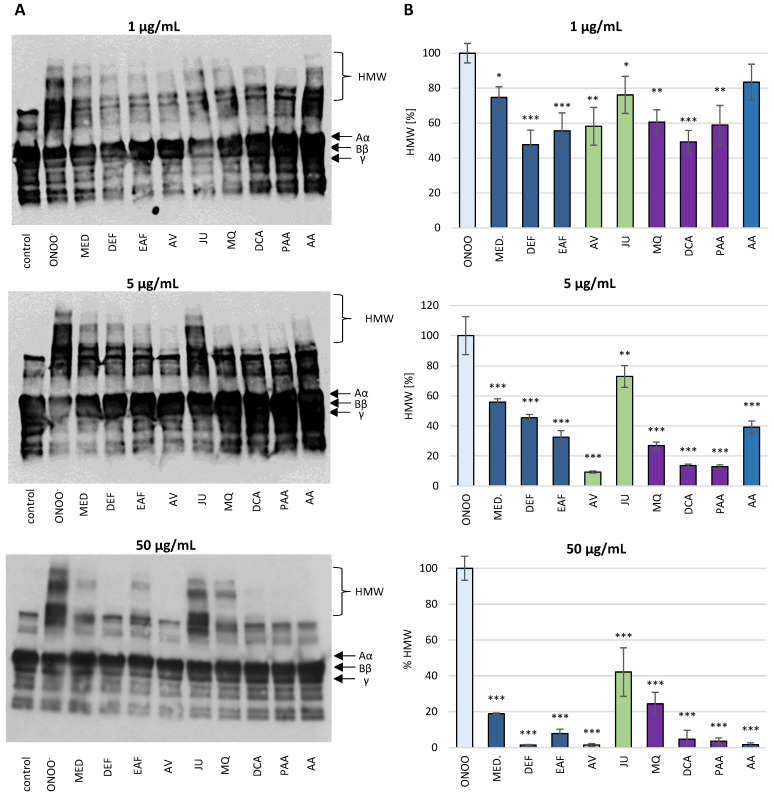
The effects of *P. spinosa* flower extracts, their native model constituents and potential in vivo metabolites on ONOO^−^-induced modifications of fibrinogen in blood plasma. The immunodetection of fibrinogen among the blood plasma proteins was carried out by the western blot (WB) method, using the anti-fibrinogen antibody. The figure represents a typical WB pattern of fibrinogen bands detected by the anti-fibrinogen antibody in blood plasma, being a result of the alternative splicing of this protein, its multistep post-translational modification and physiological proteolysis in blood circulation. The protein bands localized under the fibrinogen gamma chain correspond to the presence of partial degradation products of this protein, occurring under physiological conditions. The chemiluminescence was recorded into X-ray films. (**A**) The representative blots of three independent experiments. (**B**) Relative intensities of HMW aggregates’ bands detected in the fibrinogen in plasma matrix. The intensities of the HMW aggregates’ bands in samples treated with ONOO^−^ in the absence of the analytes was assumed as 100%. Results are presented as means ± SE (*n* = 3). Statistical differences: * *p* < 0.05, ** *p* < 0.01, and *** *p* < 0.001 for ONOO^−^-treated samples in the presence of the analytes (1, 5, 50 µg/mL) versus ONOO^−^-treated samples in the absence of the analytes. For the bars’ colors, see Figure 3.

Following the experiments on the isolated fibrinogen, the protective effect of the analytes on the ONOO^−^-induced modifications of fibrinogen were tested in blood plasma using the western blot method with an anti-fibrinogen antibody (Figure 9). The exposure of the human blood plasma samples to ONOO^−^ influenced the plasma fibrinogen and resulted in alterations of its structure. The western blot pattern of fibrinogen in the control (untreated) blood plasma revealed bands corresponding to the fibrinogen Aα, Bβ and γ chains, as well as lower bands corresponding to fibrinogen degradation products, naturally circulating in the blood. As in the experiments on the isolated protein, in the ONOO^−^-treated blood plasma chemiluminescence was also emitted by the bands corresponding to the HMW aggregates, which appeared on top of the blot.

The pre-incubation of human blood plasma with the investigated analytes (1–50 µg/mL) evidently reduced the extent of the ONOO^−^-induced modifications of the structure of plasma fibrinogen (Figure 9). A dose-dependent reduction of the chemiluminescence intensity of bands corresponding to HMW aggregates was observed. Furthermore, the activity of the blackthorn flower extracts, model native compounds and metabolites was higher or comparable to the positive standard (AA).

## 4. Discussion

ONOO^−^ is a short-lived oxidant species generated in vivo by the diffusion-controlled reaction of nitric oxide (NO) and a superoxide radical (O_2_^•^^−^). Although not a free radical in nature, ONOO^−^ is much more reactive than its progenitors and is able to attack and damage most critical biomolecules, including proteins and lipids [6,32,33]. The mechanism of this action may cover the direct reaction of ONOO^−^ via one- or two-electron oxidation processes with crucial moieties in proteins including thiols, iron/sulfur centers and zinc fingers. Moreover, ONOO^−^ may indirectly mediate oxidation by decomposing into highly reactive ROS such as hydroxyl (^•^OH) and nitrogen dioxide (^•^NO_2_) radicals [32,33]. Both ONOO^−^ and all of its secondary radicals may react with various amino acids in the peptide chain and consequently alter the protein structure and function [6]. One of the most prevalent reactions is tyrosine nitration, a two-step process in which a hydrogen atom is first abstracted from tyrosine to produce a tyrosyl radical that next promptly reacts with ^•^NO_2_ to generate 3-NT [34]. Furthermore, ONOO^−^ may trigger lipid peroxidation in membranes, liposomes and lipoproteins, resulting in the generation of lipid hydroperoxyl radicals, conjugated dienes and aldehydes [32,33].

The present work is a continuation and extension of our previous research [18], which pointed out the ability of *P. spinosa* flower extracts to protect human plasma components and to normalize/enhance the NEAC of plasma under the oxidative/nitrative stress conditions induced by ONOO^−^. As oxidative/nitrative modifications of plasma proteins and lipids are observed in patients with various CVDs [3,7,35], *P. spinosa* flower extracts seem to be promising in the context of cardiovascular protection. However, some aspects concerning the reported biological activity remained unrecognized, especially the contribution of individual phenolics to the extracts’ effects in plasma. Thus, in the first part of the current study, the antioxidant capacity of the eight model constituents of blackthorn flowers was investigated and juxtaposed with the activity of MED in the same previously applied in vivo relevant model of human plasma under ONOO^−^-induced oxidative stress conditions. The extract MED was selected for the study as a representative of the overall phenolic matrix of the flowers and an equivalent of the most popular medicinal preparations produced from the plant material, such as tinctures and liquors [18]. As the phenolic matrix of blackthorn flowers shows a high complexity [18,20,21], among the selected native polyphenols were representatives of different groups of compounds, including flavonoid aglycones (KA and QU), flavonoid monoglycosides (JU and AV), flavonoid diglycosides (KT), procyanidins (PA2), caffeoylquinic acids (CHA) and simple hydroxycinnamic acids (*p*-CA) (Figure 1).

In our experiments on a human plasma model, the plasma samples were exposed to ONOO^−^ at the concentration of 100–150 µM (depending on the test) for 5 min. The applied concentrations of ONOO^−^ caused measurable changes in the levels of the analyzed oxidative stress markers. Moreover, they corresponded to the levels of ONOO^−^ available in vivo in specific compartments and sites of inflammation, including the cardiovascular system [32]. The literature evidence indicated that a bolus addition of 250 µM ONOO^−^ to the experimental system corresponded to a physiologically achievable level of 1 µM ONOO^−^, maintained for 7 min [36]. The results indicated that the polyphenolic constituents of the blackthorn flowers have beneficial effects on the antioxidant status of plasma and protect the plasma proteins and lipids against oxidative/nitrative modifications with an effectiveness similar or higher than that of the source extract MED (Figure 3). Previously, we have reported a significant correlation between the antioxidant effects of the flower extracts in plasma and their phenolic levels [18]. All these together suggest that the low molecular weight polyphenols are crucial for the activity of MED. As was mentioned above, the investigated *P. spinosa* polyphenols should be considered as representatives of the major groups of phytochemicals present in the flower extracts. For example, JU has been selected as a model structure for KA monoglycosides. However, according to our previous research [20,21], the blackthorn extracts also contained the relevant levels of KA 3-*O*-*β*-D-xylopyranoside, KA 3-*O*-*α*-L-rhamnopyranoside and KA 7-*O*-*α*-L-rhamnopyranoside. Likewise, AV is a model of QU glycosides, among which the considerable levels of QU 3-*O*-*β*-D-xylopyranoside, QU 3-O-*α*-L-rhamnopyranoside and QU 3-*O*-*α*-L-arabinopyranoside were also observed. Similarly, in the case of caffeoylquinic acids, apart from the tested CHA, its isomers’ neochlorogenic acid and cryptochlorogenic acid are also present in the extracts [20,21]. Among the analyzed polyphenols, QU, KA, AV, CHA and PA2 were the most efficient antioxidants, and the contribution of the compounds’ fractions they represented to the activity of the extracts might be relevant. Nonetheless, the contents of these constituents in the extracts should also be taken into account. High activity parameters obtained for QU and AV, as well as the abundance of QU derivatives in the extracts, might suggest their prominent contribution to the activity of the extracts. The findings from the present study are generally consistent with our previous report, which pointed out the strong scavenging capacity of QU, AV, PA2 and CHA toward multiple in vivo relevant oxidants (ONOO^−^, O_2_^•^^−^, NO^•^, HO^•^, H_2_O_2_, and HClO) [19]. However, the current investigation revealed that KA and its glycosides, JU and KT (compounds prevalent in blackthorn flowers), displayed a noticeable antioxidant potential in the protection of human plasma components under ONOO^−^-induced stress. Interestingly, these constituents were previously proved to be incapable of effectively scavenging ONOO^−^ [19]. Thus, the mechanism behind the observed protective effects of KA and its derivatives on the human plasma model was probably unrelated to a direct reaction with ONOO^−^, but refers rather to the scavenging of other ROS operating in plasma under the applied experimental conditions, including ONOO^−^-derived secondary radicals formed in the induced chain reactions. The accumulating evidence of the antiradical effects of polyphenols against CO_3_^•–^and ^•^NO_2_ [37], as well as the efficiency of KA derivatives in HO^•^ scavenging confirmed in our previous experiment [19], support these assumptions.

Strong evidence from large observational epidemiological studies pointed out the association between various CVDs and hemostatic abnormalities [38,39]. The alterations in physiology of the hemostatic system might be a result of the oxidative and nitrative modifications of its crucial components. One of the molecules crucial for hemostasis and highly susceptible to oxidant attack is fibrinogen, constituting about 4% of plasma proteins [6,7,40]. Regarding the structure, it is a 340-kDa hexameric glycoprotein comprised of two sets of disulfide-bridged three non-identical chains termed Aα, Bβ and γ [7,41]. The main function of fibrinogen is the formation of the fibrin network. In the executive step of hemostatic clot formation, fibrinogen is converted by thrombin to fibrin monomers that are subsequently polymerized to insoluble fibrin. Additionally, fibrinogen plays an important role in platelet aggregation [7]. The oxidative modifications of the fibrinogen molecule are attributed to a specific reaction with amino acid residues, such as 3-NT formation, dityrosine crosslinking and tryptophan oxidation [6,7]. The abovementioned structural changes can influence the function of fibrinogen and lead to the formation of dysfunctional hemostatic clots. In particular, the exposition of fibrinogen to ONOO^−^ might result in the altered kinetics of fibrin formation, including decreased fibrin polymerization, increased lag time and decreased maximum absorbance during clot polymerization assays. Moreover, the structure and biomechanical properties of fibrin might be modified. For example, a slower plasmin degradation of fibrin clots has been reported. Furthermore, the impairment of platelet adhesion and aggregation was also observed [6,7,40,42].

Due to the importance of fibrinogen for the hemostatic balance of the blood, the second part of the present work involved the study of the potential impact of *P. spinosa* analytes on the structure of this protein under the oxidative stress conditions caused by ONOO^−^. The investigation included MED and its fractions DEF and EAF, which, according to our previous research, seem to be the most advantageous for a wider application and closer investigation [18,19,20,21]. The fractions DEF and EAF are highly concentrated complexes of polyphenols obtained by a liquid-liquid partitioning of MED with the TPC levels of 464.6 mg gallic acid equivalents (GAE)/mg dw and 584.1 mg GAE/g dw, respectively [18]. The use of DEF and EAF enabled an observation of all possible effects of blackthorn phenolics on the target protein. Moreover, to identify the compounds that are crucial for the activity of the extracts, the analyses included two selected native compounds (JU and AV), the most abundant polyphenols in blackthorn flowers. The results revealed significant structural changes in the fibrinogen molecule after the exposure to ONOO^−^, mainly resulting from the oxidation and/or nitration of amino acid residues, including tyrosine and tryptophan. The modifications included the increased formation of 3-NT, the decreased volumes of Aα, Bβ and γ chains, and the presence of additional bands corresponding to HMW aggregates, that might be partly explained by the conversion of tyrosine residues to dityrosine. A significant decrease of tryptophan fluorescence after the exposition of fibrinogen to ONOO^−^ was also revealed, which is consistent with the literature data that suggests the high susceptibility of this amino acid to oxidative modifications [43]. Furthermore, in accordance with previous reports [6,9], the different vulnerabilities of fibrinogen subunits to oxidative and/or nitrative modifications were observed. In particular, the Aα chain was the most prone to changes, while the Bβ and γ chains were more resistant. This might be connected with the conformation of the fibrinogen molecule—the Bβ and γ chains are highly intertwined in a tertiary structure with very little free polypeptide, while the Aα domains form polar structures exposed on the surface of the fibrinogen molecule [6,9].

The pre-incubation with *P. spinosa* flower extracts and model native constituents effectively protected fibrinogen against the harmful action of ONOO^−^. The strongest effects were observed toward 3-NT and HMW aggregates formation, which suggest the particular efficacy of the analytes in the protection of tyrosine residues. On the other hand, the influence of the analytes on tryptophan oxidation was slightly lower. It is well established that the oxidative and/or nitrative modifications of the fibrinogen structure, consequently disturbing its functions, are closely linked with numerous CVDs [7,9,40,42]. For instance, elevated levels of nitrated fibrinogen were documented in subjects with coronary artery disease, ischemic stroke and venous thromboembolism [7,9]. Thus, some of the expected health benefits of *P. spinosa* extracts might be associated with their protective activity toward undesired modifications of fibrinogen. Interestingly, one of the major constituents of the extracts, JU (kaempferol 3-*O*-*α*-L-arabinofuranoside), showed the lowest effectiveness in all tests, while the second tested model constituent, AV (quercetin 3-*O*-*α*-L-arabinofuranoside), displayed a high effectiveness. As these compounds differ only in the presence of one hydroxyl group (JU: one hydroxyl group at 4′ position; AV: two hydroxyl groups at 3′ and 4′ positions), the results demonstrated the strong influence of the *ortho*-dihydroxyl structure of polyphenols on their antioxidant activity. This is consistent with our previous study, that revealed the importance of *ortho*-dihydroxyl substitution for the scavenging capacity toward in vivo relevant oxidants and the lack of scavenging activity of JU toward ONOO^−^ [19]. The literature data on the protective effects of polyphenols and polyphenolic plant materials toward fibrinogen is limited. Bijak et al. [12] reported that the commercial extract of *Aronia melanocarpa* berries with a concentration of 0.5–50 µg/mL significantly diminished tyrosine nitration and the formation of HMW aggregates caused by the exposition to ONOO^−^. The inhibition of 3-NT formation was also observed in the case of (–)-epicatechin by about 23% and 40% at the concentrations of 1 and 10 µM (0.29 and 2.9 µg/mL), respectively [13]. These results are comparable to the ones we obtained for AV, which decreased the 3-NT formation by about 33% and 76% at the concentration of 1 and 5 µg/mL, respectively.

*P. spinosa* flower extracts demonstrated a significant antioxidant potential in both human plasma and isolated fibrinogen models, and the effects were comparable or even superior to those of pure polyphenols. It should be emphasized that the phenolic fraction constitutes only a part of the dry weight of the extracts. For example, for MED, the total phenolic content (TPC) determined previously by the Folin–Ciocalteu method was 206.07 ± 10.86 mg GAE/g dw [18], while the content of the analytes quantified by the HPLC-PDA method (TPH) was 157.47 mg/g dw [21]. Consequently, the concentration of MED in plasma samples at 5 µg/mL corresponds to a phenolic level of, at most, 1.03 µg/mL. Thus, the extract effects were higher than expected, which might probably be attributed to some synergistic effects of its individual constituents. It is often observed that polyphenol-rich extracts bring significant advantages over pure compounds, as they provide synergistic activity [44]. The synergy has been reported for different classes of polyphenols, including those present in *P. spinosa* flower extracts (flavonols, flavan-3-ols and quinic acid pseudodepsides). For example, the study on epicatechin (ECA), quercitrin (QCT) and CHA mixed in various proportions showed that almost all used combinations exerted some synergistic effects in the O_2_^•−^ scavenging test. The highest increase in antioxidant activity was demonstrated for QCT and CHA in the proportion 1:2 µg/mL, where the effect was about 30% higher than the expected theoretical value [45]. The other study concerning the interaction between ECA, CHA and 3-*O*-(2″-*β*-D-xylopyranosyl)-*β*-D-galactopyranoside (QXG) in a FRAP assay revealed that the tested compounds exhibited different effects, depending on the combination. In the case of ECA and CHA the synergistic activity was the highest and observed regardless of the concentration ratio, while the synergy between CHA and QXG was observed only if the flavonoid prevailed in the mixture [46]. Additionally, some non-phenolic constituents of the extracts might enhance the activity of polyphenols in plasma and partly contribute to the extracts’ effects. For instance, it was revealed that amino acids may act synergistically with phenolic compounds even when they do not display the antioxidant activity themselves [47].

The protective effects of blackthorn flower extracts and model compounds toward plasma components, including fibrinogen under oxidative stress conditions, were significant at concentrations as low as 1–5 µg/mL. Taking into account the results of the bioavailability studies, these levels may possibly be reached for polyphenols in plasma after oral supplementation [22,48,49]. For example, it has been reported that after the consumption of 150 mg of pure isoquercitrin (QU 3-glucoside), the QU plasma concentration amounted to 5 µM, which is equivalent to 1.5 µg/mL [50]. The studies evaluating the bioavailability of KA and its glycosides are limited. However, it has been suggested that the bioavailability of KA is higher than that of QU due to the elevated lipophilicity [49]. Therefore, the lower concentration levels (1–5 µg/mL) used in the present study appear to closely correspond to the range of physiological levels available for polyphenols in vivo after the ingestion of polyphenol-rich dietary products.

On the other hand, it is generally recognized that, after oral administration, polyphenols may undergo extensive metabolization during their passage through the human organism, and their overall biological effect is also related to the products of biotransformation. After oral intake, only free phenolic acids, flavonoid aglycones and some glucosides—as well as, to some extent, simple flavan-3-ols or proanthocyanidin dimers—can be directly absorbed in the small intestine. After absorption, they are conjugated to glucuronides, sulfates and methyl derivatives in the gut mucosa and inner tissues. However, the majority of dietary polyphenols, such as esterified phenolic acids and most flavonoid glycosides or proanthocyanidins with a higher degree of polymerization reach the colon, where they are extensively metabolized by the gut microbiota into a wide array of low molecular weight phenolic acids [25,51,52,53]. For instance, the primary gut microbiota metabolites of flavonols including QU and KA glycosides are DCA, PAA and PCA [51]. The proanthocyanidins are transformed into PPA, 3-(3′-hydroxyphenyl)propionic acid, 4-hydroxyphenylacetic acid and phenylvalerolactone [52], while CHA is first hydrolyzed by the gut microbiota to caffeic acid and next transformed into derivatives of phenylpropionic acids, such as DCA and PPA [53]. Thus, polyphenols can be found in plasma in both their native intact form (if absorbed unmodified) and as metabolites. Due to this fact, for a more thorough evaluation of the expected in vivo effects, the analyses on both models of human plasma and isolated fibrinogen also included compounds considered to be the main phenolic metabolites of blackthorn extracts. They were selected based on the phytochemical profile of *P. spinosa* flowers and the available literature data [51,52,53]. Among the investigated metabolites were MQ (the primary product of QU conjugation during the second phase of metabolism), as well as DCA, PCA, PAA and PPA, which are the degradation products of flavonols, flavanols and caffeoylquinic acids by the gut microbiota (Figure 2).

Phenolic metabolites effectively protected human plasma components, including fibrinogen, against ONOO^−^-induced damage, and their activity in some tests was higher than that of native polyphenols. The products of the gut microbiota transformation, such as DCA and PAA, displayed a remarkable efficiency in both models. The number of bioactivity studies of conjugated derivatives and microbial metabolites of polyphenols has increased in the last few years, and their results also point to the beneficial effects of some of these analytes, particularly as antioxidant, anti-inflammatory and anticancer agents [24,25]. The significant antioxidant activity of the metabolites was also highlighted in our previous work, regarding the scavenging capacity toward in vivo operating ROS [19]. The metabolic biotransformation of polyphenols can generally lead to new properties or to the loss of parental activity. Nevertheless, most studies revealed, like ours, that the metabolites retain or enhance the beneficial effects of their precursors [25]. Moreover, the metabolites derived from microbial biotransformation show superior bioavailability parameters compared to those of their native compounds. In particular, they might reach higher maximal concentration levels and stay in circulation longer [25,54]. Thus, the metabolites might play an important role in biological pathways in the human body, acting as signaling, stimulatory and inhibitory molecules, and potentially contribute to the prevention of various diseases, including CVDs [25]. A similar or higher intrinsic biological potential, as well as greater concentrations of polyphenolic metabolites than their precursors in the circulation and tissue compartments, might explain the low bioavailability/high bioactivity paradox reported for polyphenols. Despite the relatively low plasmatic concentrations reached in vivo, polyphenolic compounds were shown to be responsible for many biological effects [24,25].

It is worth noting that the activity of *P. spinosa* flower extracts, pure polyphenols and phenolic metabolites was comparable or superior to that of AA, which is the most important water-soluble antioxidant in human plasma. It is well established that AA can effectively neutralize ROS in vivo, regenerate other low molecular weight antioxidants (α-tocopherol, glutathione, urate and β-carotene) from their free radical forms, as well as inhibit the oxidation of LDL [55]. On the other hand, when the plasma AA concentration is below the optimal value of 50 µM (8.8 µg/mL), the redox hemostasis is altered, which might be one of the pre-conditions for the development and progression of oxidative stress-related diseases [56]. In this context, *P. spinosa* flower polyphenols exhibiting high antioxidant efficiency at the concentrations that can be achieved in vivo after oral administration might support the endogenous non-enzymatic antioxidant system and reduce the negative consequences of the disturbed redox status.

## 5. Conclusions

The present paper is the first evaluation of the potential protective activity of *P. spinosa* flower extracts toward fibrinogen under oxidative stress conditions and provides insight into the role of native and metabolized polyphenols as contributory factors to this activity. The results revealed that the extracts at in vivo relevant levels considerably reduced the structural changes in the fibrinogen molecule caused by the harmful action of ONOO^−^. In particular, they diminished the oxidation and nitration of amino acid residues, including tyrosine and tryptophan, and the formation of HMW aggregates. The low molecular weight polyphenols were shown to be crucial to the protective effects of the extracts toward fibrinogen and other plasma components. As the demonstrated capacities might be considered as some of the mechanisms behind the maintenance of the hemostatic balance of blood, *P. spinosa* flower extracts seem to be promising candidates for use in the prophylaxis or adjunctive therapy of CVDs associated with hemostatic abnormalities. Nevertheless, their real effects should be verified in vivo in animal and clinical studies, after the necessary toxicity tests. Moreover, the superior activity profile observed for MED in comparison to the native constituents suggests the existence of some synergistic effects between phenolics, which should be investigated in future studies. Furthermore, the potential impact of the non-phenolic compounds (which might also be present in MED) on the extract activity should be evaluated, as these constituents might display synergies with phenolics even when they do not possess antioxidant activity themselves. Since the non-phenolic profile of the blackthorn flower extracts remains unexplored, the metabolomic analysis is required to identify the other constituents of the extracts.

## Figures and Tables

**Table 1:** Quantitative standardization data for *P. spinosa* flower dry extracts used in the present study.

Phytochemical Content	MED	DEF	EAF	References
**TPC (mg GAE/g dw)**	206.07 ± 10.86 ^a^	464.57 ± 20.57 ^b^	584.07 ± 12.98 ^c^	[18]
**TFC (mg/g dw)**	125.12 ± 0.55 ^a^	490.63 ± 8.16 ^c^	325.53 ± 4.23 ^b^	[18]
**TPA (mg CYE/g dw)**	45.13 ± 2.38 ^a^	49.5 ± 2.23 ^a^	109.43 ± 3.71 ^b^	[18]
**TAC (mg/g dw)**	29.24 ± 0.76 ^c^	8.76 ± 0.27 ^a^	17.20 ± 0.47 ^b^	[18]
**TPH (mg/g dw)**	157.47 ^a^	491.69 ^c^	353.07 ^b^	[20,21]

Results are presented as mean values ± SD (*n* = 3) calculated per dry weight (dw) of the extracts. Different superscripts (a–c) in each row indicate significant differences in the means (*p* < 0.05). TPC—total phenolic content quantified by the Folin–Ciocalteu method; TFC—total flavonoid content determined by HPLC-PDA as the content of flavonoid aglycones after acid hydrolysis of glycosides; TPA—total proanthocyanidin content quantified by the *n*-butanol-HCl method; TAC—total content of phenolic acids determined by HPLC-PDA; TPH—total phenolic content determined by the HPLC-PDA-fingerprint method. For detailed quantitative profiles, see [18,20,21].

## Data Availability

Not applicable.

## Supplementary Materials

The following are available online at https://www.mdpi.com/article/10.3390/antiox10040581/s1, Table S1: Content of the main groups of phytochemicals present in *P. spinosa* flower extracts and their representatives investigated in the current study according to Marchelak et al. [20,21], Table S2: Correlation coefficients (*r*) and probability (*p*) values of linear relationships between methods used to evaluate the potential effects of the blackthorn flower extracts, model native polyphenols and phenolic metabolites on fibrinogen under oxidative stress conditions.

## Abbreviations

3-NT	3-nitrotyrosine
AA	ascorbic acid
AV	avicularin (quercetin 3-*O*-α-L-arabinofuranoside
C-ELISA	the competitive enzyme-linked immunosorbent assay
CHA	chlorogenic acid
CVDs	cardiovascular diseases
CYE	Cyanidin chloride equivalents
DCA	3-(3′,4′-dihydroxyphenyl)propionic acid (dihydrocaffeic acid)
DEF	diethyl ether fraction
dw	dry weight
EAF	ethyl acetate fraction
GAE	Gallic acid equivalents
H_2_O_2_	hydrogen peroxide
HOCl	hypochlorous acid
HMW	high molecular weight
JU	juglanin (kaempferol 3-*O*-α-L-arabinofuranoside)
KA	kaempferol
KT	kaempferitrin (kaempferol 3,7-di-*O*-α-L-rhamnopyranoside)
LOOH	lipid hydroperoxides
MED	defatted methanol-water (7:3, *v*/*v*) extract
MQ	miquelianin (quercetin 3-*O*-β-D-glucuronopyranoside)
MW	molecular weight
NEAC	non-enzymatic antioxidant capacity of plasma
NO^•^	nitric oxide
ONOO^−^	peroxynitrite
O_2_^•−^	superoxide anion
OH^•^	hydroxyl radical
PA2	proanthocyanidin A2
PAA	2-(3′,4′-dihydroxyphenyl)acetic acid
*p*-CA	*p*-coumaric acid
PCA	protocatechuic acid
PPA	3-(4′-hydroxyphenyl)propionic acid
QU	quercetin
ROS	reactive oxygen species
SDS-PAGE	sodium dodecyl sulfate-polyacrylamide gel electrophoresis
TAC	total content of phenolic acids (HPLC-PDA)
TBARS	thiobarbituric acid-reactive substances
TFC	total flavonoid content (HPLC-PDA)
TPA	total proanthocyanidin content (*n*-butanol/HCl assay)
TPC	total phenolic content (Folin-Ciocalteu assay)
TPH	total phenolic content (HPLC-PDA-fingerprint)
