# Supplementary material for: The Effects of Prunus spinosa L. Flower Extracts, Model Polyphenols and Phenolic Metabolites on Oxidative/Nitrative Modifications of Human Plasma Components with Particular Emphasis on Fibrinogen In Vitro
Source: Antioxidants (Basel). 2021 Apr 9;10(4):581. doi: 10.3390/antiox10040581 (PMC8069707; doi:10.3390/antiox10040581)
Supplement: Supplementary file 1 [file antioxidants-10-00581-s001.pdf]

Supplementary materials:

The Effects of *Prunus spinosa* L. Flower Extracts, Model Polyphenols and Phenolic Metabolites on Oxidative/Nitrative Modifications of Human Plasma Components with Particular Emphasis on Fibrinogen In Vitro

Anna Marchelak [1,*], Joanna Kolodziejczyk-Czepas [2], Paulina Wasielewska [2], Pawel Nowak [2] and Monika A. Olszewska [1]

1 Department of Pharmacognosy, Faculty of Pharmacy, Medical University of Lodz, 90-151 Lodz, Poland; monika.olszewska@umed.lodz.pl
2 Department of General Biochemistry, Faculty of Biology and Environmental Protection, University of Lodz, 90-236 Lodz, Poland; joanna.kolodziejczyk@biol.uni.lodz.pl (J.K.-C.); paulinakapusta94@wp.pl (P.W.); pawel.nowak@biol.uni.lodz.pl (P.N.)
* Correspondence: anna.marchelak@umed.lodz.pl

Table S1. Content of the main groups of phytochemicals present in *P. spinosa* flower extracts and their representatives investigated in the current study according to Marchelak et al. [20,21].

Group of compounds	Content [mg/g dw]			Representatives	Content [mg/g dw]		
	MED	DEF	EAF		MED	DEF	EAF
**Flavonoid aglycones**	2.38	84.00	30.27	**QU**	1.32 ± 0.06	42.92 ± 1.09	20.99 ± 0.50
				**KA**	1.06 ± 0.01	41.08 ± 1.15	9.28 ± 0.34
**Quercetin monoglycosides**	28.81	101.65	94.48	**AV**	14.89 ± 0.65	71.04 ± 2.42	28.81 ± 1.12
**Kaempferol monoglycosides**	33.24	270.18	79.79	**JU**	13.73 ± 0.43	96.14 ± 1.33	16.90 ± 0.26
**Kaempferol diglycosides**	58.35	14.45	162.74	**KT**	17.42 ± 0.79	6.13 ± 0.30	41.46 ± 0.19
**Procyanidins**	9.53	29.79	48.66	**PA2**	-	-	-
**Caffeoylqunic acids**	24.36	-	10.83	**CHA**	5.64 ± 0.11	-	5.69 ± 0.32
**Hydroxycinnamic acids**	-	8.24	-	***p*-CA**	-	8.24 ± 0.22	-

Results are presented as means ± SD ($n = 3$). For details see [20,21].

Table S2. Correlation coefficients (r) and probability (p) values of linear relationships between methods used to evaluate the potential effects of the blackthorn flower extracts, model native polyphenols and phenolic metabolites on fibrinogen under oxidative stress conditions.

***r* (*p*) for:**	**SDS-PAGE**	**WB anty-3NT**	**3-NT ELISA**	**Trp oxid.**	**WB anty-FG**
**SDS-PAGE**		0.9135 (0.000)***	0.9331 (0.000)***	0.5808 (0.001)***	0.8950 (0.000)***
**WB anty-3NT**	0.9135 (0.000)***		0.9673 (0.000)***	0.6175 (0.000)***	0.9455 (0.000)***
**3-NT ELISA**	0.9331 (0.000)***	0.9673 (0.000)***		0.6411 (0.000)***	0.9522 (0.000)***
**Trp oxid.**	0.5808 (0.001)***	0.6175 (0.000)***	0.6411 (0.000)***		0.5944 (0.001)***
**WB anty-FG**	0.8950 (0.000)***	0.9455 (0.000)***	0.9522 (0.000)***	0.5944 (0.001)***	

Asterisks indicate statistical significance of the estimated linear relationships (* $p < 0.05$, ** $p < 0.01$, and *** $p < 0.001$).
